# *catena*-Poly[[[tetra­aqua­zinc(II)]-μ-4,4′-bipyridine-κ^2^ *N*:*N*′] benzene-1,4-di­carboxyl­ate]

**DOI:** 10.1107/S1600536809019412

**Published:** 2009-06-06

**Authors:** Ming-Bo Ruan, Jian-Cheng Deng, Zhi-Gang Li, Jing-Wei Xu

**Affiliations:** a College of Chemistry, Xiangtan University, Hunan 411105, People’s Republic of China; b National Analytical Research Center of Electrochemistry and Spectroscopy, Changchun Institute of Applied Chemistry, Chinese Academy of Sciences, Changchun 130022, People’s Republic of China; c State Key Laboratory of Electroanalytical Chemistry, Changchun Institute of Applied Chemistry, Chinese Academy of Sciences, Changchun 130022, People’s Republic of China

## Abstract

In the title compound, {[Zn(C_10_H_8_N_2_)(H_2_O)_4_](C_8_H_4_O_4_)}_*n*_, the Zn^II^ atoms, lying on a twofold rotation axis, are bridged by 4,4′-bipyridine ligands, resulting in a linear chain along the *b* axis. In the chain, the Zn^II^ atom adopts a slightly distorted octa­hedral coordination geometry involving four water mol­ecules at the equatorial positions. The noncoordinated benzene-1,4-dicarboxyl­ate anion, which is also located on a twofold rotation axis, bridges adjacent chains through O—H⋯O hydrogen bonds, forming a three-dimensional supra­molecular network.

## Related literature

For background information on hydro­thermal reactions, see: Yaghi *et al.* (2003 ▶). For hydrogen-bond graph-set motifs, see: Bernstein *et al.* (1995 ▶).
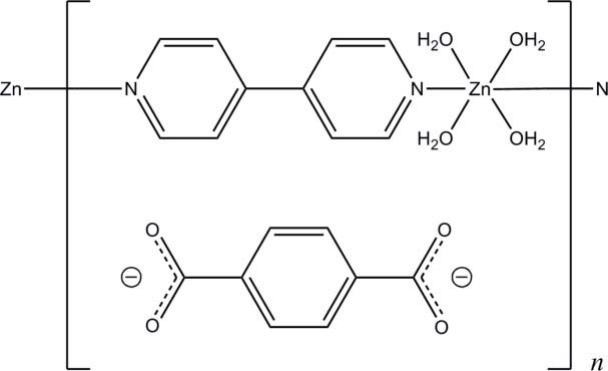


## Experimental

### 

#### Crystal data

[Zn(C_10_H_8_N_2_)(H_2_O)_4_](C_8_H_4_O_4_)
*M* *_r_* = 457.75
Monoclinic, 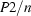

*a* = 6.9861 (12) Å
*b* = 11.3436 (19) Å
*c* = 11.3219 (19) Å
β = 101.209 (3)°
*V* = 880.1 (3) Å^3^
*Z* = 2
Mo *K*α radiation
μ = 1.45 mm^−1^
*T* = 186 K
0.21 × 0.18 × 0.12 mm

#### Data collection

Bruker APEX CCD area-detector diffractometer
Absorption correction: multi-scan (**SADABS**; Sheldrick, 1996 ▶) *T*_min_ = 0.751, *T*_max_ = 0.845
4812 measured reflections
1735 independent reflections
1465 reflections with *I* > 2σ(*I*)
*R*_int_ = 0.035

#### Refinement

*R*[*F* ^2^ > 2σ(*F* ^2^)] = 0.042
*wR*(*F* ^2^) = 0.108
*S* = 1.08
1735 reflections
136 parameters
H-atom parameters constrained
Δρ_max_ = 0.64 e Å^−3^
Δρ_min_ = −0.27 e Å^−3^

### 

Data collection: *SMART* (Bruker, 1998 ▶); cell refinement: *SAINT-Plus* (Bruker, 2003 ▶); data reduction: *SAINT-Plus*; program(s) used to solve structure: *SHELXS97* (Sheldrick, 2008 ▶); program(s) used to refine structure: *SHELXL97* (Sheldrick, 2008 ▶); molecular graphics: *SHELXTL* (Sheldrick, 2008 ▶); software used to prepare material for publication: *SHELXTL*.

## Supplementary Material

Crystal structure: contains datablocks global, I. DOI: 10.1107/S1600536809019412/is2415sup1.cif

Structure factors: contains datablocks I. DOI: 10.1107/S1600536809019412/is2415Isup2.hkl

Additional supplementary materials:  crystallographic information; 3D view; checkCIF report

## Figures and Tables

**Table 1:** Hydrogen-bond geometry (Å, °)

*D*—H⋯*A*	*D*—H	H⋯*A*	*D*⋯*A*	*D*—H⋯*A*
O1—H1*AA*⋯O3^i^	0.87	1.89	2.753 (3)	169
O1—H1*AB*⋯O4^ii^	0.86	2.08	2.893 (3)	157
O2—H2*AA*⋯O4^iii^	0.85	1.87	2.718 (3)	173
O2—H2*AB*⋯O3^iv^	0.84	1.91	2.742 (3)	171

Symmetry codes: (i) 
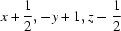
; (ii) 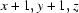; (iii) 
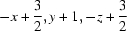
; (iv) 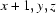.

**Fig. 1.**
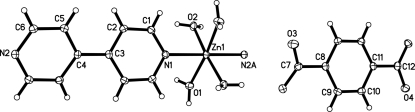
The structure of (I), showing the atomic numbering scheme. Displacement ellipsoids are drawn at the 30% probability level.

**Fig. 2.**
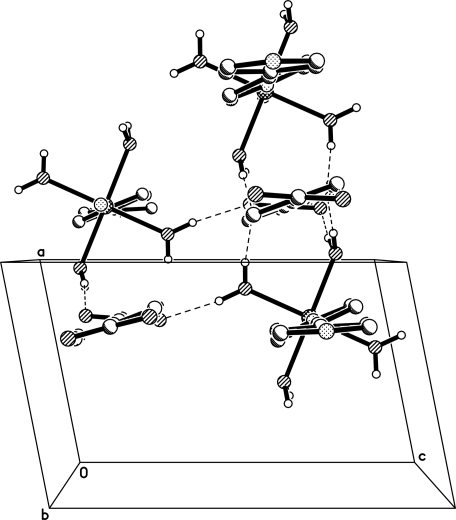
View of the three-dimensional supramolecular structure in (I). Dashed lines indicate hydrogen bonds.

## supplementary crystallographic information

### Comment

Hydro(solvo)thermal reaction has shown a kind of promising technique for the preparation of complexes with novel structures and special properties (Yaghi *et al.*, 2003). Here we report the structure of the title compound, (I), which contains one-dimensional cation chains and non-coordinated benzene-1,4-dicarboxylate as couteranions under solvothermal condition.

Compound, (I), as shown in Fig. 1, consists of one-dimensional [Zn(C_10_H_8_N_2_)(H_2_O)_4_]_n_ cation chains and benzene-1,4-dicarboxylate anions. The Zn(II) atoms are in a slightly distorted octahedral geometry, where two N atoms from two 4,4'-bipyridine ligands occupy the axial positions, and the equatorial positions are completed by four water molecules. The compound crystallizes in a centrosymmetric space group, which defines twofold axes along both the one-dimensional chains and the benzene-1,4-dicarboxylate anions.

In the crystal structure, intermolecular O—H···O hydrogen bonds are present (Table 1 and Fig. 2) and the coordinated water molecules are linked with two benzene-1,4-dicarboxylate anions to form *R*_4_^4^(12) and *R*_6_^4^(16) hydrogen-bonding rings (Bernstein *et al.*, 1995). In addition, there are strong π···π interactions between pyridine rings and phenyl rings at (*x*, *y*, *z*) and (1/2 - *x*,1 + *y*, 3/2 - *z*), with the shortest atom-to-center distance of 3.322 (4) Å. The two kinds of interactions lead to a three-dimensional supramolecular network (Fig. 2).

### Experimental

Compound (I) was solvothermally prepared from a reaction mixture of Zn(BF_4_)_2_ (0.2 mmol), 4,4'-bipyridine (0.1 mmol), benzene-1,4-dicarboxylic acid (0.1 mmol), methanol (3 ml) and distilled water (8 ml) in a molar ratio of 2:1:740:4444; the pH value was adjusted to 4.8 with trimethylamine and acetic acid. The mixture was stirred for 20 min at room temperature and then sealed in a Teflon-lined stainless steel autoclave with a 23 ml capacity at 423 K for 72 h. After cooling to room temperature, colourless block-shaped crystals were obtained; these were washed with deionized water, filtered, and dried in air (yield 48% based on Zn).

### Refinement

C-bound H atoms were placed geometrically (C—H = 0.93 Å) and were refined using a riding model, with *U*_iso_(H) = 1.2*U*_eq_(C). H atoms on the water molecules were located in a difference Fourier map and the positions were fixed, with *U*_iso_(H) = 1.2*U*_eq_(O).

### Crystal data

**Table:**

[Zn(C_10_H_8_N_2_)(H_2_O)_4_](C_8_H_4_O_4_)	*F*(000) = 472
*M**_r_* = 457.75	*D*_x_ = 1.727 Mg m^−^^3^
Monoclinic, *P*2/*n*	Mo *K*α radiation, λ = 0.71073 Å
Hall symbol: -P 2yac	Cell parameters from 1213 reflections
*a* = 6.9861 (12) Å	θ = 2.3–24.8°
*b* = 11.3436 (19) Å	µ = 1.45 mm^−^^1^
*c* = 11.3219 (19) Å	*T* = 186 K
β = 101.209 (3)°	Block, colorless
*V* = 880.1 (3) Å^3^	0.21 × 0.18 × 0.12 mm
*Z* = 2	

### Data collection

**Table:**

Bruker APEX CCD area-detector diffractometer	1735 independent reflections
Radiation source: fine-focus sealed tube	1465 reflections with *I* > 2σ(*I*)
graphite	*R*_int_ = 0.035
φ and ω scans	θ_max_ = 26.0°, θ_min_ = 1.8°
Absorption correction: multi-scan (*SADABS*; Sheldrick, 1996)	*h* = −8→4
*T*_min_ = 0.751, *T*_max_ = 0.845	*k* = −14→12
4812 measured reflections	*l* = −12→13

### Refinement

**Table:**

Refinement on *F*^2^	Primary atom site location: structure-invariant direct methods
Least-squares matrix: full	Secondary atom site location: difference Fourier map
*R*[*F*^2^ > 2σ(*F*^2^)] = 0.042	Hydrogen site location: inferred from neighbouring sites
*wR*(*F*^2^) = 0.108	H-atom parameters constrained
*S* = 1.08	*w* = 1/[σ^2^(*F*_o_^2^) + (0.0572*P*)^2^ + 0.2035*P*] where *P* = (*F*_o_^2^ + 2*F*_c_^2^)/3
1735 reflections	(Δ/σ)_max_ = 0.001
136 parameters	Δρ_max_ = 0.64 e Å^−^^3^
0 restraints	Δρ_min_ = −0.27 e Å^−^^3^

### Special details

**Table:**

Geometry. All e.s.d.'s (except the e.s.d. in the dihedral angle between two l.s. planes) are estimated using the full covariance matrix. The cell e.s.d.'s are taken into account individually in the estimation of e.s.d.'s in distances, angles and torsion angles; correlations between e.s.d.'s in cell parameters are only used when they are defined by crystal symmetry. An approximate (isotropic) treatment of cell e.s.d.'s is used for estimating e.s.d.'s involving l.s. planes.
Refinement. Refinement of *F*^2^ against ALL reflections. The weighted *R*-factor *wR* and goodness of fit *S* are based on *F*^2^, conventional *R*-factors *R* are based on *F*, with *F* set to zero for negative *F*^2^. The threshold expression of *F*^2^ > σ(*F*^2^) is used only for calculating *R*-factors(gt) *etc*. and is not relevant to the choice of reflections for refinement. *R*-factors based on *F*^2^ are statistically about twice as large as those based on *F*, and *R*- factors based on ALL data will be even larger.

### Fractional atomic coordinates and isotropic or equivalent isotropic displacement parameters (Å^2^)

**Table:**

	*x*	*y*	*z*	*U*_iso_*/*U*_eq_	
Zn1	0.7500	0.60167 (4)	0.7500	0.0231 (2)	
N1	0.7500	0.7908 (3)	0.7500	0.0219 (8)	
N2	0.7500	1.4169 (3)	0.7500	0.0233 (8)	
O1	0.8792 (3)	0.61311 (19)	0.59227 (19)	0.0310 (6)	
H1AA	0.8253	0.6201	0.5164	0.037*	
H1AB	0.9941	0.6430	0.6054	0.037*	
O2	1.0343 (3)	0.59315 (16)	0.8530 (2)	0.0272 (5)	
H2AA	1.1223	0.6455	0.8544	0.033*	
H2AB	1.1024	0.5339	0.8452	0.033*	
O3	0.2410 (4)	0.38845 (18)	0.8474 (2)	0.0329 (6)	
O4	0.2079 (3)	−0.22784 (18)	0.64951 (18)	0.0285 (5)	
C1	0.8163 (4)	0.8531 (3)	0.8503 (3)	0.0231 (7)	
H1A	0.8638	0.8121	0.9211	0.028*	
C2	0.8179 (4)	0.9744 (3)	0.8544 (3)	0.0227 (7)	
H2A	0.8642	1.0131	0.9267	0.027*	
C3	0.7500	1.0391 (4)	0.7500	0.0192 (9)	
C4	0.7500	1.1695 (3)	0.7500	0.0177 (9)	
C5	0.7615 (5)	1.2339 (3)	0.8561 (3)	0.0223 (7)	
H5	0.7694	1.1949	0.9292	0.027*	
C6	0.7612 (5)	1.3551 (3)	0.8525 (3)	0.0255 (7)	
H6	0.7691	1.3963	0.9243	0.031*	
C7	0.2500	0.3377 (4)	0.7500	0.0245 (10)	
C8	0.2500	0.2043 (4)	0.7500	0.0201 (9)	
C9	0.1895 (4)	0.1414 (3)	0.6432 (3)	0.0225 (7)	
H9	0.1501	0.1818	0.5711	0.027*	
C10	0.1878 (4)	0.0195 (3)	0.6440 (3)	0.0225 (7)	
H10	0.1442	−0.0210	0.5724	0.027*	
C11	0.2500	−0.0438 (4)	0.7500	0.0200 (9)	
C12	0.2500	−0.1761 (4)	0.7500	0.0244 (10)	

### Atomic displacement parameters (Å^2^)

**Table:**

	*U*^11^	*U*^22^	*U*^33^	*U*^12^	*U*^13^	*U*^23^
Zn1	0.0313 (4)	0.0149 (3)	0.0225 (3)	0.000	0.0040 (2)	0.000
N1	0.027 (2)	0.0174 (18)	0.0222 (19)	0.000	0.0068 (15)	0.000
N2	0.028 (2)	0.0169 (18)	0.0242 (19)	0.000	0.0038 (16)	0.000
O1	0.0362 (14)	0.0349 (13)	0.0221 (12)	−0.0073 (10)	0.0066 (10)	−0.0017 (9)
O2	0.0287 (13)	0.0174 (11)	0.0339 (13)	−0.0010 (9)	0.0017 (10)	−0.0001 (9)
O3	0.0464 (16)	0.0256 (12)	0.0253 (13)	0.0101 (10)	0.0036 (11)	−0.0026 (9)
O4	0.0399 (14)	0.0217 (12)	0.0236 (12)	0.0017 (10)	0.0057 (10)	−0.0039 (9)
C1	0.0257 (18)	0.0225 (15)	0.0208 (15)	0.0014 (13)	0.0037 (13)	0.0027 (12)
C2	0.0265 (19)	0.0234 (16)	0.0179 (16)	−0.0005 (13)	0.0034 (13)	−0.0030 (12)
C3	0.018 (2)	0.019 (2)	0.023 (2)	0.000	0.0075 (18)	0.000
C4	0.012 (2)	0.018 (2)	0.023 (2)	0.000	0.0030 (17)	0.000
C5	0.0253 (17)	0.0227 (16)	0.0186 (15)	−0.0007 (13)	0.0032 (13)	0.0014 (12)
C6	0.033 (2)	0.0228 (15)	0.0202 (16)	−0.0001 (14)	0.0046 (14)	−0.0012 (12)
C7	0.025 (3)	0.022 (2)	0.024 (2)	0.000	−0.0009 (19)	0.000
C8	0.017 (2)	0.021 (2)	0.023 (2)	0.000	0.0079 (18)	0.000
C9	0.0266 (19)	0.0230 (15)	0.0181 (15)	0.0028 (13)	0.0047 (13)	0.0030 (12)
C10	0.0215 (18)	0.0267 (16)	0.0195 (16)	0.0009 (13)	0.0040 (13)	−0.0017 (12)
C11	0.018 (2)	0.020 (2)	0.023 (2)	0.000	0.0089 (18)	0.000
C12	0.023 (2)	0.022 (2)	0.029 (2)	0.000	0.006 (2)	0.000

### Geometric parameters (Å, °)

**Table:**

Zn1—N2^i^	2.096 (3)	C3—C2^ii^	1.394 (3)
Zn1—O2^ii^	2.101 (2)	C3—C4	1.479 (6)
Zn1—O2	2.101 (2)	C4—C5	1.395 (3)
Zn1—N1	2.145 (3)	C4—C5^ii^	1.395 (3)
Zn1—O1	2.156 (2)	C5—C6	1.376 (4)
Zn1—O1^ii^	2.156 (2)	C5—H5	0.9300
N1—C1	1.342 (3)	C6—H6	0.9300
N2—C6	1.344 (3)	C7—O3^iii^	1.256 (3)
O1—H1AA	0.8719	C7—C8	1.513 (6)
O1—H1AB	0.8571	C8—C9	1.397 (3)
O2—H2AA	0.8525	C8—C9^iii^	1.397 (3)
O2—H2AB	0.8379	C9—C10	1.383 (4)
O3—C7	1.256 (3)	C9—H9	0.9300
O4—C12	1.263 (3)	C10—C11	1.394 (3)
C1—C2	1.377 (4)	C10—H10	0.9300
C1—H1A	0.9300	C11—C10^iii^	1.394 (4)
C2—C3	1.394 (3)	C11—C12	1.500 (6)
C2—H2A	0.9300	C12—O4^iii^	1.263 (3)
			
N2^i^—Zn1—O2^ii^	87.36 (5)	C3—C2—H2A	120.0
N2^i^—Zn1—O2	87.36 (5)	C2^ii^—C3—C2	116.4 (4)
O2^ii^—Zn1—O2	174.73 (10)	C2^ii^—C3—C4	121.79 (19)
N2^i^—Zn1—N1	180.000 (1)	C2—C3—C4	121.79 (19)
O2^ii^—Zn1—N1	92.64 (5)	C5—C4—C5^ii^	116.8 (4)
O2—Zn1—N1	92.64 (5)	C5—C4—C3	121.58 (18)
N2^i^—Zn1—O1	93.45 (6)	C5^ii^—C4—C3	121.58 (18)
O2^ii^—Zn1—O1	92.63 (9)	C6—C5—C4	119.9 (3)
O2—Zn1—O1	87.69 (9)	C6—C5—H5	120.1
N1—Zn1—O1	86.55 (6)	C4—C5—H5	120.1
N2^i^—Zn1—O1^ii^	93.45 (6)	N2—C6—C5	123.1 (3)
O2^ii^—Zn1—O1^ii^	87.69 (9)	N2—C6—H6	118.4
O2—Zn1—O1^ii^	92.63 (9)	C5—C6—H6	118.4
N1—Zn1—O1^ii^	86.55 (6)	O3—C7—O3^iii^	125.5 (4)
O1—Zn1—O1^ii^	173.10 (12)	O3—C7—C8	117.3 (2)
C1—N1—C1^ii^	116.4 (4)	O3^iii^—C7—C8	117.3 (2)
C1—N1—Zn1	121.81 (18)	C9—C8—C9^iii^	118.5 (4)
C1^ii^—N1—Zn1	121.81 (18)	C9—C8—C7	120.73 (19)
C6^ii^—N2—C6	117.2 (4)	C9^iii^—C8—C7	120.73 (19)
C6^ii^—N2—Zn1^iv^	121.42 (18)	C10—C9—C8	120.5 (3)
C6—N2—Zn1^iv^	121.42 (18)	C10—C9—H9	119.8
Zn1—O1—H1AA	130.6	C8—C9—H9	119.8
Zn1—O1—H1AB	114.1	C9—C10—C11	121.3 (3)
H1AA—O1—H1AB	110.2	C9—C10—H10	119.4
Zn1—O2—H2AA	125.3	C11—C10—H10	119.4
Zn1—O2—H2AB	118.2	C10^iii^—C11—C10	117.9 (4)
H2AA—O2—H2AB	98.0	C10^iii^—C11—C12	121.03 (19)
N1—C1—C2	123.7 (3)	C10—C11—C12	121.03 (19)
N1—C1—H1A	118.2	O4—C12—O4^iii^	124.6 (4)
C2—C1—H1A	118.2	O4—C12—C11	117.7 (2)
C1—C2—C3	119.9 (3)	O4^iii^—C12—C11	117.7 (2)
C1—C2—H2A	120.0		

Symmetry codes: (i) *x*, *y*−1, *z*; (ii) −*x*+3/2, *y*, −*z*+3/2; (iii) −*x*+1/2, *y*, −*z*+3/2; (iv) *x*, *y*+1, *z*.

### Hydrogen-bond geometry (Å, °)

**Table:**

*D*—H···*A*	*D*—H	H···*A*	*D*···*A*	*D*—H···*A*
O1—H1AA···O3^v^	0.87	1.89	2.753 (3)	169
O1—H1AB···O4^vi^	0.86	2.08	2.893 (3)	157
O2—H2AA···O4^vii^	0.85	1.87	2.718 (3)	173
O2—H2AB···O3^viii^	0.84	1.91	2.742 (3)	171

Symmetry codes: (v) *x*+1/2, −*y*+1, *z*−1/2; (vi) *x*+1, *y*+1, *z*; (vii) −*x*+3/2, *y*+1, −*z*+3/2; (viii) *x*+1, *y*, *z*.

## References

[bb1] Bernstein, J., Davis, R. E., Shimoni, L. & Chang, N.-L. (1995). *Angew. Chem. Int. Ed. Engl.* **34**, 1555–1573.

[bb2] Bruker (1998). *SMART* Bruker AXS Inc., Madison, Wisconsin, USA.

[bb3] Bruker (2003). *SAINT-Plus* Bruker AXS Inc., Madison, Wisconsin, USA.

[bb4] Sheldrick, G. M. (1996). *SADABS* University of Göttingen, Germany.

[bb5] Sheldrick, G. M. (2008). *Acta Cryst.* A**64**, 112–122. 10.1107/S0108767307043930 18156677

[bb6] Yaghi, O. M., O’Keeffe, M., Ockwig, N. W., Chae, H. K., Eddaoudi, M. & Kim, J. (2003). *Nature (London)*, **423**, 705–714. 10.1038/nature01650 12802325

